# Synchrotron Radiation X-ray Diffraction Techniques Applied to Insect Flight Muscle

**DOI:** 10.3390/ijms19061748

**Published:** 2018-06-13

**Authors:** Hiroyuki Iwamoto

**Affiliations:** Japan Synchrotron Radiation Research Institute, SPring-8, 1-1-1 Kouto, Sayo-cho, Sayo-gun, Hyogo 679-5198, Japan; iwamoto@spring8.or.jp; Tel.: +81-791-58-2506

**Keywords:** insect flight muscle, X-ray diffraction, synchrotron radiation, X-ray microbeam, time-resolved measurement

## Abstract

X-ray fiber diffraction is a powerful tool used for investigating the molecular structure of muscle and its dynamics during contraction. This technique has been successfully applied not only to skeletal and cardiac muscles of vertebrates but also to insect flight muscle. Generally, insect flight muscle has a highly ordered structure and is often capable of high-frequency oscillations. The X-ray diffraction studies on muscle have been accelerated by the advent of 3rd-generation synchrotron radiation facilities, which can generate brilliant and highly oriented X-ray beams. This review focuses on some of the novel experiments done on insect flight muscle by using synchrotron radiation X-rays. These include diffraction recordings from single myofibrils within a flight muscle fiber by using X-ray microbeams and high-speed diffraction recordings from the flight muscle during the wing-beat of live insects. These experiments have provided information about the molecular structure and dynamic function of flight muscle in unprecedented detail. Future directions of X-ray diffraction studies on muscle are also discussed.

## 1. Introduction

Like light microscopy and electron microscopy, X-ray diffraction is one of the techniques used to resolve the fine structure of objects by using the scattering of electromagnetic waves or electron beams. Generally, the spatial resolution obtained in each technique is defined by the diffraction limit, which is comparable to the wavelength of the beams used. Both X-ray beams and the electron beams (used in the electron microscope) have wavelengths comparable to or shorter than the lengths of atomic bonds and, in principle, atomic resolution should be obtained by using both of these techniques.

In protein crystallography, such atomic-resolution structures of proteins are routinely obtained. For this purpose, the generation of high-quality protein crystals is essential, but this is very often a difficult process. The resolution of protein 3-D structures obtained by single-particle analyses in cryo-electron microscopy (cryo-EM) used to be worse than 1 nm, but, recently, this has been dramatically changed by the advent of direct detectors, which have nominal pixel sizes equivalent to ~1 Å at the scale of the sample. By using this type of detector, the resolution has improved to 2 to 3 Å [1]. The processing that leads to the determination of such high-resolution structures is enormous and usually tens of thousands of particle images are collected, classified and merged.

The focus of this review is not the pursuit of such a high spatial resolution, but is on the non-invasive nature of X-ray diffraction methods that allows observation of biomolecules functioning under physiological conditions. The high penetrability of X-ray beams even allows us to observe the biomolecules in living organisms, and brilliant synchrotron radiation X-ray beams make it possible to do it at a high time resolution. In this study, we describe examples of the application of this technique to muscle, with a special emphasis on insect flight muscle, which has a structure regular enough for productive use of X-ray diffraction recordings. First, the basic principles of X-ray diffraction are briefly described. Next, we describe the instruments needed to record X-ray diffraction patterns especially in synchrotron radiation facilities. Then we describe the latest results of research obtained by using this technique and, lastly, we discuss some future direction for X-ray diffraction studies on muscle.

## 2. Outline of X-ray Diffraction Principles

As for detailed explanation for the principles of X-ray diffraction and its application to muscle, a number of excellent textbooks and reviews have been published [2,3,4,5,6,7,8,9,10,11,12,13,14] and the readers who are interested in learning the principles in depth are encouraged to refer to these articles.

### 2.1. Scattering of X-rays by An Object

As in the case of visible light, X-ray photons are scattered by an object. In the atoms that constitute the object, the nuclei (protons and neutrons) have little contribution to the scattering. As such, the scattering by an object is almost equivalent to the scattering by the electrons within the object. Mathematically, the distribution of the scattered photons is the Fourier transform of the object and this Fourier transform is called the structure factor of the object. To be accurate, the intensity of the scattering is proportional to the square of the amplitude of the structure factor.

The length in the structure factor is inversely related to the length in the world that we normally observe, which is why the space for the structure factor is called reciprocal space. Reciprocal space is intuitively difficult to comprehend. However, if one performs a Fourier transformation once again (inverse Fourier transformation), the function in the reciprocal space is brought back to real space. The lenses of light and electron microscopes do this directly and the real-space image of the object is restored at a different magnification.

The Fourier transform of the object or the structure factor is a complex number and has a real part and an imaginary part. In other words, it has an amplitude and a phase. To restore the real-space image by inverse Fourier transformation, information of both the amplitude and the phase is required. With the lenses used in light microscopes and electron microscopes, the two quantities are preserved and the real-space image of the object can be restored.

In the case of X-rays, lenses are not usually used. The intensity distribution of the scattered waves (diffraction pattern) is recorded by various types of detectors, and the patterns are directly analyzed in various ways. Regardless of the type of detectors, they can record only the amplitude of the scattered waves, and the phase information is lost. Without phase information, the real-space image of the object cannot be restored. This is called the phase problem and great efforts have been made to solve the problem in every field of X-ray diffraction studies including protein crystallography.

Why are lenses not used for X-ray? The reason is that it is difficult (although not impossible) to fabricate lenses that can be used to create an X-ray microscope. The reasons for this difficulty are as follows: (1) the refractive index of matter for X-rays is very close to 1 (actually very slightly less than 1) so refractive X-ray lenses are not feasible. (2) as for mirrors the condition in which total reflection occurs is very limited for X-rays (the angle of incidence to the mirror must be very small) so that concave mirrors that are used for telescopes cannot be fabricated for X-rays. (3) even if we can fabricate focusing optics for X-rays, the accuracy of fabrication must be at the atomic scale to obtain the spatial resolution expected from the wavelengths of X-rays.

**Figure 1:**
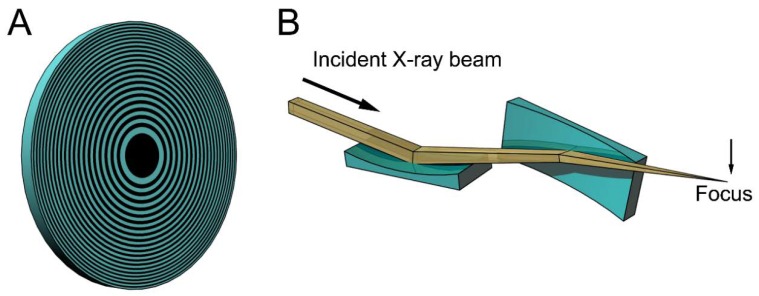
Schematic drawings of some focusing devices for X-ray beams. (**A**) Fresnel zone plate. This is a circular grating with alternating densities. The focal distance depends on the X-ray wavelengths and white X-rays do not focus; (**B**) Kirkpatric-Baez (KB) mirrors. These are most commonly used for focusing X-ray beams in synchrotron radiation facilities, but are rarely used as imaging optics. The focal distance does not depend on the X-ray wavelengths and can be changed by altering the bend of the mirrors. The drawings are exaggerated.

Figure 1 shows examples of the focusing devices designed for X-rays. The device in Figure 1A is called a Fresnel zone plate, and is usually fabricated by micro-lithography or nano-lithography. It is a circular grating consisting of concentric rings of alternating transparent and scattering zones, and the interval between the two neighboring rings are closer as they approach the outer edge. Due to this configuration, the beams scattered by the outer zones are diffracted more strongly towards the center. Therefore, all the diffracted beams are directed towards a single focus. X-ray microscopes using this device have actually been tested (e.g., [15,16,17]). The spatial resolution is determined by the accuracy of lithography and it is currently around 20 nm. This value is rather disappointing when compared to the wavelengths of X-rays (~0.1 nm) and is only slightly better than that of super-resolution light microscopes [18]. Another type of focusing device is a pair of bent planar mirrors called Kirkpatric-Baez (KB) mirrors (see Figure 1B). An X-ray microscope using this device is free of chromatic aberration that is inherent in the zone plates [19]. The spatial resolution obtained with this X-ray microscope is 50 nm, and this also depends on the accuracy of fabrication of the mirrors. X-ray microscopes are still in the developmental stage and it will take years to reach a practical stage for biological use.

There is a third type of lens that can be used for X-ray. It uses a computer as a lens. This type of imaging is called the lens-less imaging, and this emerging technique will be briefly explained at the end of this review.

### 2.2. Analysis of Scattered X-rays in the Reciprocal Space

In protein crystallography, the phase information is restored by heavy-atom labeling, multi-wavelength anomalous diffraction (MAD) or single-wavelength anomalous diffraction (SAD) methods [20]. In non-crystalline diffraction studies including fiber diffraction and protein solution scattering, the scattered intensities are usually and traditionally directly analyzed and interpreted in reciprocal space. This means that researchers are requested to “read” the diffraction pattern as it is. For detailed explanation as for “how to read” readers are encouraged to refer to the textbooks and reviews listed in the first paragraph of this section, since only the basics (for application to muscle) are described here.

First, a diffraction pattern (the square of the structure factor of the object) represents the spectrum of density plotted against the spatial frequency known as the power spectrum. For an object with continuous densities (such as a protein molecule), its power spectrum is continuous. If there are regularly repeating structures, it means that the densities prevail at a certain spatial frequency (or interval) and show up as a peak in the power spectrum. Therefore, a diffraction pattern is sensitive to periodic structures.

Usually an object is three-dimensional, which means its structure factor is also three-dimensional. When its diffraction pattern is recorded by using a planar detector, one can record only one slice of its structure factor. To collect all the data needed to reconstruct the 3-D structure of the object, it must be rotated. In the case of a muscle specimen, however, all the intensities of the structure factor are usually recorded in a single exposure without the need of rotating the specimen. This is because a muscle or a muscle fiber contains a large number of myofibrils with random orientations around their long axes. This situation makes the diffraction pattern rotationally averaged, and it poses some difficulty in reconstructing the un-rotated sarcomeric structure.

A useful and important notion in “reading” a diffraction pattern is called the convolution theorem. The convolution of two functions, *f*(*t*) and *g*(*t*), is expressed in the following equation.

(1) $$c(t)=\int_{-\infty }^{\infty }f(\tau )g(t-\tau )d\tau$$

**Figure 2:**
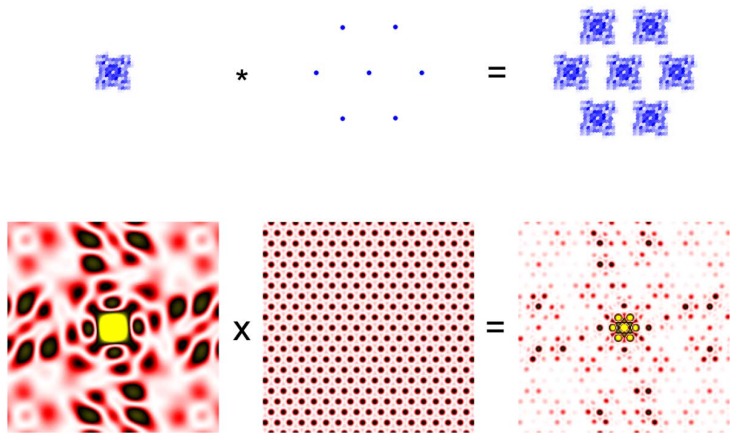
Convolution theorem. A small crystal of protein molecules in which the molecules are arranged in a hexagonal lattice (**Top right**) is the convolution of two functions. The function defining the electron density of each molecule (**Top left**) and the function defining the hexagonal lattice (**Top middle**). The asterisk represents the operation of convolution. The bottom row represents the diffraction pattern (Fourier transform) of the functions in the top row. The diffraction pattern from the protein crystal is the product of the pattern from a single molecule from the lattice. The protein is meant to be a ryanodine receptor.

It is easier to understand the meaning of the equation by looking at the illustration in Figure 2. Then the convolution theorem states that the Fourier transformation of the convolution of two functions is the product of the Fourier transforms of the two functions.

The basic structure of the sarcomere of a striated muscle, either vertebrate or insect, is the two sets of myofilaments (actin and myosin) arranged in a hexagonal lattice. Like DNA molecules, the monomers of the actin and myosin filaments are helically arranged and they give rise to very similar diffraction patterns. The theory of diffraction from a helix was established in 1950s by F. Crick and other researchers [21]. They demonstrated that the Fourier transform of a helix is described as a series of Bessel functions of the first kind. In a two-dimensional diagram, the Bessel functions take the form of a series of layer-like features arranged in an X-shape. Therefore, each of the layer-like reflections is called a layer-line reflection. The helical pitch of the actin filament is different from that of the myosin filament so that the two filaments give rise to layer-line reflections at different positions in the diffraction pattern (although a few of them overlap with each other).

In the usual configuration of diffraction recording from muscle specimens, the long axis of the muscle fiber is made perpendicular to the axis of the incident X-ray beam. In the diffraction pattern recorded in this way, two axes can be defined. One is perpendicular to the fiber axis and is called the equator. The reflections that arise along the equator are called equatorial reflections. Equatorial reflections are the strongest of all reflections from muscles and are dominated by the contribution from the hexagonal lattice of the filaments.

**Figure 3:**
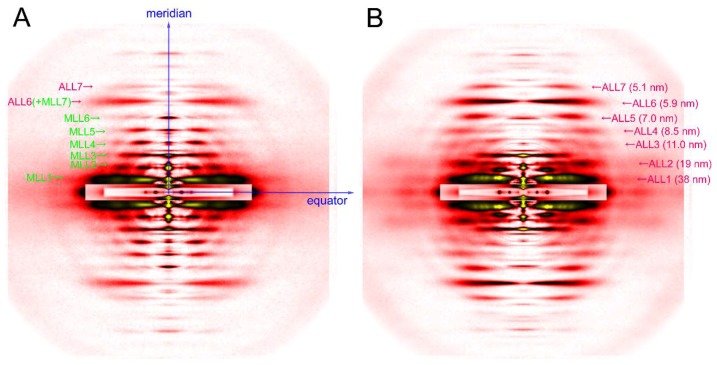
Diffraction pattern recorded from rabbit skeletal muscle fibers. (**A**) relaxed; (**B**) rigor. The patterns were recorded from an array of 30 skinned single fibers of psoas muscle. Note that a series of myosin layer-line reflections (MLL, green letters) in the relaxed pattern are replaced by a series of actin layer-line reflections (ALL, purple letters).

The axis along the fiber is called the meridian. There are periodical structures along the myofilaments such as troponin, and their periodicity is reflected in reflections on the meridian. They are called meridional reflections. The helices of the myosin filaments and actin filaments are discontinuous, i.e., they also exhibit periodicities along the filament axis. Therefore, there are a number of meridional reflections arising from the monomers of actin and myosin. The reflections not on either the meridian or the equator are the layer-line reflections, which arise from the helical arrangement of actin and myosin filaments. Two typical 2-dimensional diffraction patterns from rabbit skeletal muscle fibers are shown in Figure 3. One is taken in the relaxed state (see Figure 3A) and, except for the first, sixth, and seventh actin layer-line reflections, the myosin layer-line reflections dominate, which reflects a good helical order of the helix of myosin heads. The other is taken in rigor (see Figure 3B) and the myosin layer-line reflections are replaced by numerous actin layer-line reflections. This indicates that the myosin heads attach to actin in a stereospecific manner, following the helical symmetry of actin so that the mass that contributes to the actin layer-line reflections is increased. At the same time, the intensities of the two major equatorial reflections (1,0 and 1,1) are reversed, which reflects the movement of mass from the myosin filaments to the actin filaments. In this way, one can obtain information about the molecular behavior within muscle fiber by directly interpreting the diffraction pattern.

### 2.3. What is Made Possible by the Advent of Synchrotron Radiation

The diffraction patterns seen in Figure 3 are called static diffraction patterns recorded under constant conditions of the specimen with detectors without using the capacity of time-resolved recordings (traditionally, X-ray films). Before the days of synchrotron radiation facilities, laboratory X-ray sources were used in combination with low-sensitivity (by today’s standard) X-ray films. The strongest laboratory X-ray source was a rotating-anode X-ray generator but, even with this, it took a whole day to record a diffraction pattern equivalent to the ones shown in Figure 3 from a whole sartorius muscle from a frog.

**Figure 4:**
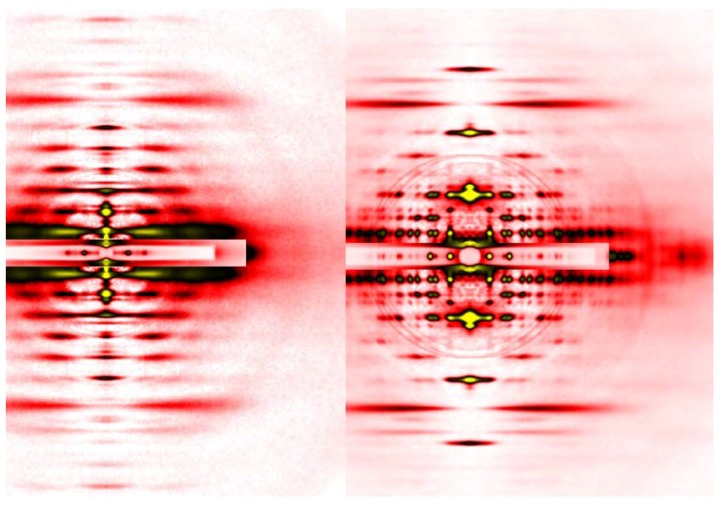
Comparison of diffraction patterns from vertebrate skeletal muscle fibers (**left**, identical to the one in Figure 3A) and insect flight muscle fibers (**right**, from giant waterbug *Lethocerus*).

The advantage of synchrotron radiation X-ray beams, especially those from so-called third-generation synchrotron radiation facilities, is their overwhelming intensity, and in terms of brilliance (a unit expressing the brightness of beams), the X-ray beams from a beamline equipped with an undulator and a monochromator is 10^8^ times brighter than those emitted from a rotating-anode generator (in the case of SPring-8). An undulator is a device inserted in the orbit of electrons in the synchrotron ring and can generate strong and highly oriented X-ray beams. An undulator consists of a series of magnets with alternating polarities, which makes the electron orbit literally undulate. Each time the electron orbit is bent, strong radiation is generated. The diffraction patterns in Figure 3 were recorded in one of such beamlines, BL45XU of SPring-8 in Japan [22], which can deliver 10^12^ photons/s. The detectors available today are much more sensitive than X-ray films and they include Imaging Plates, CCD (charge-coupled device) or CMOS (complementary metal oxide silicon)-type semiconductor detectors that either detect X-ray photons directly or are used in combination with image intensifiers or phosphor plates followed by fiber optics. By using such detectors, one can record diffraction patterns as seen in Figure 4 in a matter of a few seconds from a set of ~30 single muscle fibers in which the mass of sample in the beam is much smaller than in the case of the laboratory X-ray source, using a sartorius muscle as a specimen.

By utilizing these highly bright and well-oriented synchrotron radiation X-ray beams, one can perform time-resolved diffraction recordings at a high time resolution. In addition, diffraction recording from a very small area of the specimen can be achieved using X-ray microbeams (X-ray beams with a micrometer-order diameter) as well as a combination of both. In the following sections, we outline some examples of the application of synchrotron radiation X-ray beams to insect flight muscle.

## 3. Brief Introduction to Insect Flight Muscle

The flight muscles of insects are the muscles responsible for the wing-beat. They consist of voluminous power muscles that provide the driving force of the wing-beat, and smaller steering muscles that adjust the angle of the wings so that the insects can steer themselves during flight. Most of the winged insects have two antagonistic pairs of power muscles called dorsal longitudinal muscle (DLM) and dorso-ventral muscle (DVM). They do not drive the wings directly, but they do it indirectly by deforming the thoracic exoskeleton. For this reason, they are called indirect flight muscles. They occupy most of the space of the thoracic exoskeleton and they have been the major target for X-ray diffraction studies as well as other physiological and structural studies.

Some small insects can beat their wings at very high frequencies, e.g., ~500 Hz in mosquitoes (e.g., [23,24,25]) and up to 1000 Hz in smaller midges [26]. These frequencies are not realized by the repetition of usual contraction-relaxation cycles in which each is triggered by a motor nerve impulse. These insects have asynchronous flight muscles in which the muscle undergoes autonomous oscillation while the intracellular calcium concentration is held constant by low-frequency motor nerve impulses [27,28,29,30,31,32]).

The molecular mechanism for this high-frequency autonomous oscillation has been the main interest for insect muscle researchers. The key function is stretch activation, which refers to the delayed rise of active force after an externally applied stretch [27]. Its molecular mechanism is still under investigation.

Structurally, asynchronous flight muscle is also special. The arrangement of contractile proteins is so regular that a sarcomere of asynchronous flight muscle can be regarded as a kind of protein crystal. Because of this high regularity, the lower-order layer line reflections consist of a series of discrete spots, which would appear in the diffraction pattern from a protein crystal (e.g., [33]. See also Figure 4). It is speculated that this high regularity of structure is related to the function of stretch activation [34,35]. The flight muscle fibers isolated from a giant waterbug, *Lethocerus* have been the standard material for flight muscle research and they give rise to very high-quality diffraction patterns. Its muscle fibers are long (~10 mm) and easy to mount and are also suitable for mechanical measurements. Today, however, diffraction patterns can be recorded from the flight muscles of much smaller insects, which are so small that diffraction recording from their flight muscle would have been unimaginable before the days of synchrotron radiation.

## 4. Diffraction Recordings from *Drosophila* Flight Muscle

The advent of third-generation synchrotron radiation facilities has made it possible to perform time-resolved X-ray diffraction studies on the flight muscles of *Lethocerus* and other relatively large insects at millisecond time resolutions. During the recordings, either sinusoidal oscillations [36] or step length changes [37] were applied to isolated flight muscle fibers. An important study was done by George et al. [38] who performed time-resolved X-ray diffraction recording from the flight muscle of a hawkmoth, *Manduca*, while applying sinusoidal length changes. The muscle was not isolated, but left in situ and mechanically connected between a servomotor and a force transducer. Since the muscle fibers were intact, they were activated by electrical stimulation.

The milestone achievement is Irving and Maughan’s work on the tiny fruitfly, *Drosophila melanogaster* [39]. Their work is novel not only because the diffraction was recorded from the flight muscle of such a tiny insect, but also because it was from living and beating flies (tethered flight) and the time resolution was as high as 1 ms, which was required to take snapshots of diffraction patterns during the 200-Hz wing-beat of *Drosophila*.

This high time resolution was achieved by the use of fast-rotating X-ray shutters synchronized with the wing-beat so that diffraction patterns for a certain phase of the wing-beat were excised. Later their work was further refined and more reflection spots were analyzed with better accuracy [40]. In their work, the X-ray beams were passed along the dorso-ventral axis on the midline, and diffraction patterns were recorded from DLM.

Another study of recording diffraction patterns from *Drosophila* flight muscle was done by Iwamoto et al. [41]. In this study, the time course of development of the myofibrillar structure was followed in the pupal stage. In this study, the X-ray beams were directed through the side of the thorax so that diffraction patterns were recorded from both DLM and DVM. Additionally, diffraction patterns from what was thought to be the jump muscle were also recorded. It is not a flight muscle, and from the intensity ratio of the equatorial reflection, it is considered to have a myofilament lattice structure different from that of flight muscle. The number ratio of actin to myosin filaments is 3:1 in flight muscle, but the diffraction pattern from the jump muscle is best explained if the ratio is 5:1. In this study, diffraction recordings from single myofibrils by using X-ray microbeams were also included (see below).

## 5. Sub-Millisecond Time-Resolved Diffraction Recordings from Bumblebee Flight Muscle

Each of the beamlines in the third-generation synchrotron radiation facilities, such as BL45XU of SPring-8, is usually equipped with an undulator and a monochromator. A monochromator generates highly monochromatized X-ray beams (∆λ/λ = 0.01% where λ is the wavelength) but the flux outside the bandwidth is not utilized. The high-flux BL40XU beamline of SPring-8 is special in that it has a helical undulator, which generates a helically rotating magnetic field [42]. The X-rays produced by this undulator are already substantially monochromatic and, by omitting a monochromator, the beamline achieves an X-ray flux of 10^15^ photons/s, i.e., 1000 times brighter than in BL45XU. This is because of the wider X-ray bandwidth (∆λ/λ = 2%) so that there is a tradeoff between the bandwidth and the flux.

By utilizing this exceptionally high X-ray flux, one can perform very fast time-resolved X-ray diffraction recording when fast recording detectors are used. During the beginning of the study, a 3-CCD (charge-coupled device) camera was used in BL40XU in combination with an image intensifier. The 3-CCD camera had three CCD sensor chips that were alternately used to increase the frame rate. With a full-frame size (640 × 480 pixels), a frame rate of ~290 frames/s was achieved. By using this system, a number of fast time-resolved diffraction recordings were made on muscle specimens, including the structural changes of skeletal muscle fibers after photolysis of caged ATP [43] or caged calcium [44] and an early study on the flight muscles from bumblebee and giant waterbug [37]. The problems with this detector were that its sensitivity was not very high because the light path was split to three by prisms, and there were some frame-to-frame variations because the sensitivities of the three CCD chips were not quite identical. It also required a complex timing circuit to synchronize with external events such as the UV flash for photolysis of caged compounds or mechanical stretch.

Today, the 3-CCD detector has been replaced by faster CMOS (complementary metal oxide silicon) detectors. Each of them has a single CMOS sensor chip so that there is no frame-to-frame variation. A frame rate of up to 6400–7000 frames/s is achieved with a mega-pixel frame size (1024 × 1024 pixels). Later models have even higher sensitivities. The timing circuit can also be simpler.

By using one of these fast CMOS cameras, the responses of the diffraction patterns a step stretch (complete in 1 ms) of skinned bumblebee flight muscle fibers, which were recorded at a frame rate of 2000 frames/s [45]. It is shown that the earliest changes in the diffraction pattern after stretch are the reciprocal intensification of the 1,1,1 reflection and the diminution of the 2,0,1 reflection (the numbers are the crystallographic Miller indices, which indicates which crystallographic plane the reflection comes from). By varying the experimental conditions, these intensity changes were demonstrated to occur conspicuously only when the flight muscle fibers were calcium-activated and it was suggested that the intensity changes report the structural changes that trigger stretch activation. Three-dimensional model calculations showed that the intensity changes can be explained by the deformation of myosin heads while they are weakly attached to actin and these calculations have led to a proposal that such a deformation promotes the conversion of the myosin heads from the weakly attached form to the strongly attached, force-producing form. This mechanism would explain stretch activation essential for the action of asynchronous flight muscle.

**Figure 5:**
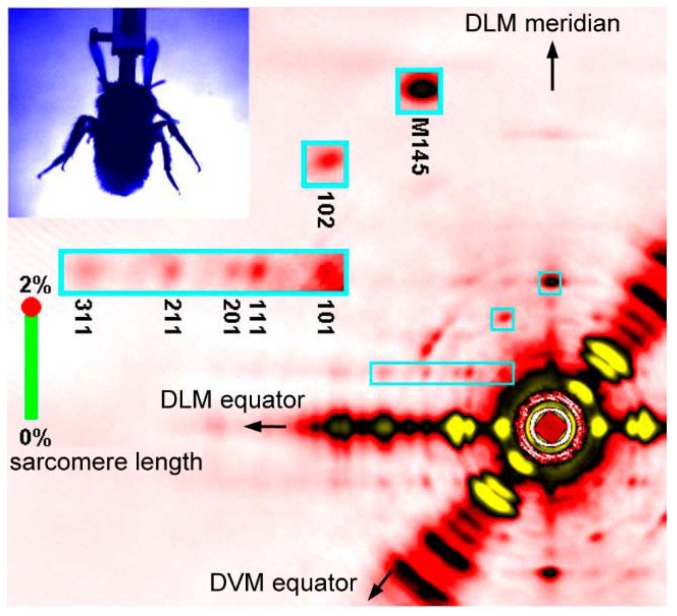
A single frame of X-ray diffraction movie recorded from the flight muscles of live, beating bumblebees (1 frame = 0.2 ms). The regions of interest (blue rectangles) are shown in a greater magnification on the left. Taken from Iwamoto & Yagi [46].

Fast diffraction recordings were also performed by using live bumblebees during tethered flight [46] (see Figure 5). Unlike the case of *Drosophila* [39,40], the X-ray beams were incident from the side of the bumblebee thorax so that the diffraction patterns of both DLM and DVM were recorded at the same time. It was shown that the two muscles act almost exactly in anti-phase with a phase shift of 180 degrees.

The frame rate was 5000 frames/s and, because the wing-beat frequencies of the bees were ~120 Hz, ~40 frames were taken for each wing beat. This frame rate made it possible to follow the time course of intensity changes during the wing-beat in detail. It was shown that the reciprocal intensity changes of the 1,1,1 and 2,0,1 reflections occur conspicuously in live bees and they occur at the right timing when the flight muscle is being stretched by its antagonistic pair. This result shows that the stretch-induced structural changes responsible for these intensity changes occur under physiological conditions in live flying bees.

## 6. Radiation Damage Issues

Although, in principle, X-ray diffraction recording is a non-invasive method to study the structure of biological specimens, irradiation caused by intense X-ray beams leads to radiation damage. This is mainly because, in an aqueous environment, free radicals are generated and they diffuse around to cause damage to biomolecules (e.g., [47]). Due to this, hydrated biological specimens are more vulnerable to radiation damage than non-hydrated specimens. In the case of the BL45XU beamline (flux = 10^12^ photons/s), radiation damage becomes evident after a few seconds of exposure. Radiation damage can be alleviated to some extent by inclusion of a reducing agent such as glutathione or dithiothreitol and catalase (e.g., ref. [48]).

Another method to reduce radiation damage is to move the specimen during exposure so that the X-ray beams do not keep hitting the same part of the specimen. This can be applied to uniform specimens. Isolated muscle fibers can be regarded as uniform along their long axis, so that they can be shifted by the distance of the beam diameter after each short exposure or they can be moved continuously during a longer exposure. The latter method is suitable for time resolved measurements. In the case of experiments using caged compounds, the full flux of the BL40XU beamline (10^15^ photons/s) was given and the fibers were moved along their long axis at the speed of 100 mm/s by using a motorized mechanical stage [37,43]. 

For recordings of static diffraction patterns, a very effective way to reduce radiation damage is to quick-freeze the specimen and keep it at a very low temperature until the exposure is finished (e.g., [49]). By doing so, biological specimens can be at least ~500 times more resistant to radiation damage than unfrozen specimens [50]. This is a very common technique for protein crystallography. If a hydrated specimen is frozen slowly, crystalline ice (hexagonal ice) is formed and this destroys the specimen because of volume expansion. To avoid this, the water in the specimen must be frozen very quickly to form amorphous (vitrified) ice. For electron microscopy, muscle fibers are often plunged onto a cooled copper block (e.g., [51]), but the vitrified layer is restricted to 10 µm or less from the plunged surface. To vitrify a greater volume of the specimen, the use of so-called cryoprotectants is very effective (e.g., [52]). These include glycerol, dimethyl sulfoxide, and 2-methyl pentanediol. These chemicals yield effective freezing results for specimens of the size of a single muscle fiber if they are used at a ~20% concentration. For comparison of various cryoprotectants and their concentrations, see Iwamoto [53].

## 7. Diffraction Recordings Using X-ray Microbeams

X-ray microbeams refer to X-ray beams with a diameter of the order of micrometers. These can be created by focusing optics such as a KB mirror and a Fresnel zone plate, which was mentioned in the section on the X-ray microscope. However, these optics usually have short focus and may not be suitable for small-angle scattering experiments such as diffraction recording from muscle. A more suitable and simple optical system is to use pinholes. Usually two or more pinholes are used in tandem. The first (upstream) pinhole defines the diameter of the microbeam (defining pinhole) and the second (downstream) pinhole cuts off the parasitic scattering generated by the edge of the first pinhole (guard pinhole). The practical lower limit of the microbeam diameter created this way is ~2 µm. If one wishes to use an aperture smaller than this, one will be forced to bring the sample very close to the defining pinhole because the beam rapidly expands to a larger diameter because of Fraunhofer diffraction.

By using X-ray microbeams, one may obtain diffraction patterns from a very confined area of the specimen. The microbeam diameter of ~2 µm is comparable to that of a single myofibril so that one is expected to be able to record a diffraction pattern from a single myofibril.

**Figure 6:**
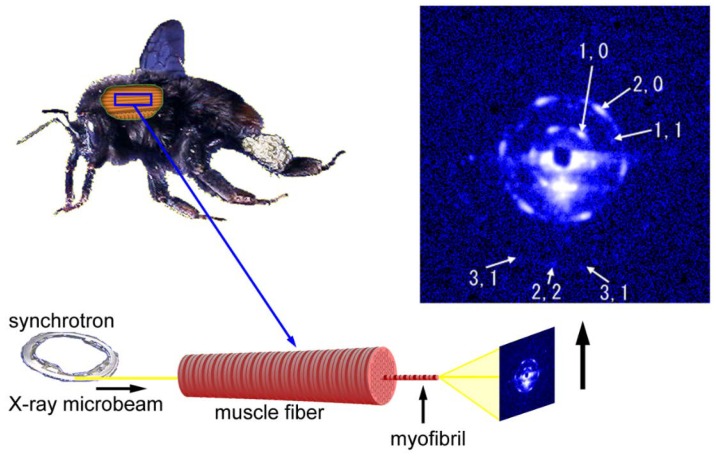
Geometry of diffraction recording (end-on configuration) from a single myofibril within a flight muscle fiber of a bumblebee by using an X-ray microbeam. The beam diameter was 2 µm and one of the first diffraction patterns recorded from a single myofibril is shown on the right. The pattern shown here is identical to the one shown in Reference [54].

The first example to record diffraction patterns from single myofibrils was done in the BL45XU beamline using bumblebee flight muscle fibers. The patterns were not recorded from isolated myofibrils. Unlike the usual configuration in fiber diffraction, the microbeams were directed along the fiber axis in an end-on configuration (see Figure 6). If the myofibrils are completely straight and the beams are completely parallel to the myofibrillar axis, then one should reasonably expect to record a pattern from a single myofibril. The results were that the recorded patterns were expected from a single hexagonal lattice of myofilaments and the reflections were arranged in a hexagonal array [54] (the Fourier transform of a hexagonal lattice is a hexagonal lattice). Therefore, the geometry of the beam and the specimen was considered to have been nearly under the ideal straight-parallel conditions as mentioned above. The myofibrils of bumblebee flight muscle are thicker than those of vertebrate skeletal muscle (diameter, ~3 µm) and are isolated from each other because of the abundance of mitochondria. These factors make it easier to record diffraction patterns from single myofibrils.

Importantly, a diffraction pattern from a single hexagonal lattice was recorded despite the fact that there were ~1000 sarcomeres in series within the beam path (the length of the specimen was ~3 mm). The length of 3 mm is close to the full length of myofibrils in the bumblebee thorax. This means that the orientations of the hexagonal lattices of successive sarcomeres are identical throughout the entire length of the myofibril. A myofibril of bumblebee flight muscle can be regarded as a single giant protein crystal.

## 8. Quick Freezing of Flight Muscle Specimens

The experiment described above was done in the BL45XU beamline by using hydrated specimens that were not quite stable or resistant to radiation damage. The quality of the diffraction patterns was not satisfactory. To improve the quality of diffraction patterns, we started to quick-freeze the specimens.

The first successful recordings from quick-frozen muscle specimens were published in 2005 [50]. Diffraction patterns were recorded from both a whole muscle fiber (of rabbit skeletal muscle) and an isolated myofibril of bumblebee in the normal configuration of recording in which the X-ray beam axis was perpendicular to the fiber axis. From the single rabbit muscle fiber, a number of layer-line reflections were clearly recorded (this is not a microbeam experiment). It is unusual that these layer-line reflections are recorded from a single muscle fiber and this is because a much longer exposure time is possible for quick-frozen specimens.

**Figure 7:**
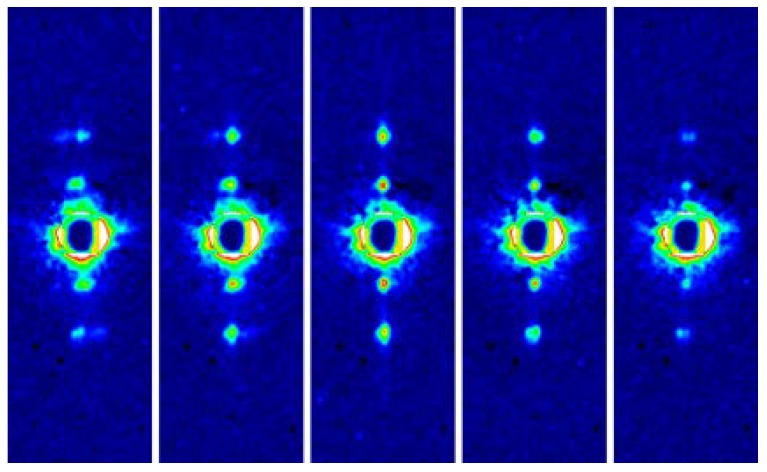
Equatorial reflections recorded from a single sarcomere within a frozen myofibril of bumblebee attached on a thin plastic film. The myofibrillar axis is in the horizontal direction. This is the color version of the figure that appeared in Reference [50].

The myofibrils from bumblebee flight muscle were affixed onto a thin plastic film before freezing. Because the beam diameter was ~2 µm, it should illuminate the area equivalent to a single sarcomere, which was ~3 µm long. The 1,0 and 2,0 equatorial reflections were clearly recorded (see Figure 7). It is again unusual that a diffraction pattern is recorded from a single sarcomere of a single myofibril and again it owes to a long exposure time allowed for frozen specimens.

## 9. Evolution of the Myofilament Structure of Winged Insects

As described in Section 7, the myofibril of bumblebee flight muscle has been shown to have a structure of a giant single protein crystal. To know whether it is true for other winged insects, we examined the lattice structure of myofibrils from a wide variety of winged insects by using the microbeam end-on diffraction method as described above. This time, the specimens were quick-frozen to improve their stability [55].

**Figure 8:**
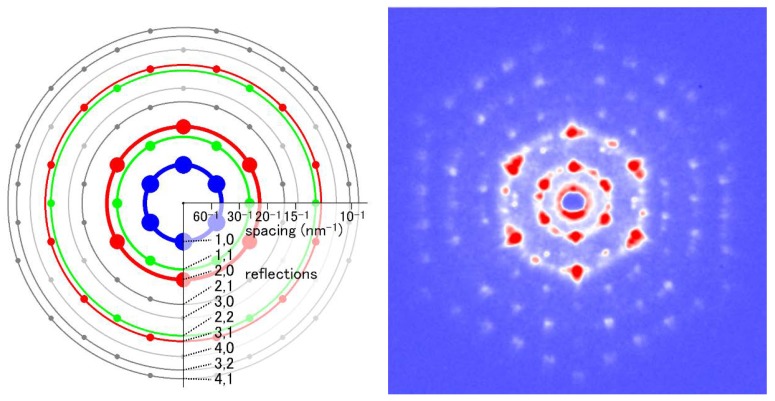
End-on diffraction pattern from a single myofibril of the flight muscle of a beetle, *Aulacophora femoralis*. The specimen was quick-frozen and the pattern was recorded at the liquid-nitrogen temperature. The left image is the schematic diagram showing the positions of the reflection spots expected from the hexagonal lattice of myofilaments. Taken from Reference [45].

Figure 8 shows one of the end-on diffraction patterns recorded from the myofibril in a frozen flight muscle fiber from a beetle, *Aulacophora femoralis*. When compared with the patterns from unfrozen specimens, the improvement of the quality of the diffraction pattern is evident, and reflections of up to 5,0 are observed. The results of end-on diffraction recording from 50 insect species showed that insect species with asynchronous flight muscles have the aforementioned giant single-crystal-like myofibrils without any exception. These insects include Diptera (flies, mosquitoes, etc.), Hymenoptera (bees, wasps, etc.), Coleoptera (beetles), and a part of Hemiptera (true bugs including giant waterbugs). Yet, insects with synchronous flight muscles generally do not have giant-single-crystal-like myofibrils. These insects include Lepidoptera (butterflies and moths), Orthoptera (grasshoppers and locusts), and many other orders. In addition, myofibrils from the flight muscle of Odonata (dragonflies and damselflies) show some single-crystal-like features.

It is surprising that the myofibrils of asynchronous flight muscles have unanimously highly ordered structure regardless of the orders to which the insects belong. This is because asynchronous flight muscles are believed to have occurred many times independently in the course of insect evolution. The universal occurrence of the giant-single-protein-crystal-like myofibrils suggests that this structure plays an essential role in the function of asynchronous flight muscle, which ensures uniform transmission of force or length changes required for the proper generation of the stretch-activated force.

## 10. Future Directions

Because the phase problem is inevitable in X-ray diffraction recordings, the right interpretation of diffraction patterns requires a proper understanding of diffraction theory. But diffraction theory is not easy to understand and this makes researchers prefer more direct methods that can directly show objects in real space.

Recently, however, we have increasing knowledge about how to preserve or recover the phase information in X-ray diffraction experiments, which is owed to advanced algorithms and improved computer performance. The techniques to restore the real-space images of the object by using this knowledge are collectively called coherent diffractive imaging (CDI) or lens less imaging [56,57,58]. To do this, coherent illumination of objects is required. Coherence refers to the property of electromagnetic waves in which the phase of the wave is the same for all the photons like in laser beams. X-ray beams with laser-like coherence are typically generated in X-ray free electron laser (XFEL) facilities, but partly coherent X-ray beams are available in the conventional synchrotron radiation facilities as well.

One of the CDI techniques is to restore lost phase information by iterative error-reducing calculations and is often called the oversampling method [59,60,61,62]. For details of the method, readers are encouraged to refer to the literature and here only an outline is described.

**Figure 9:**
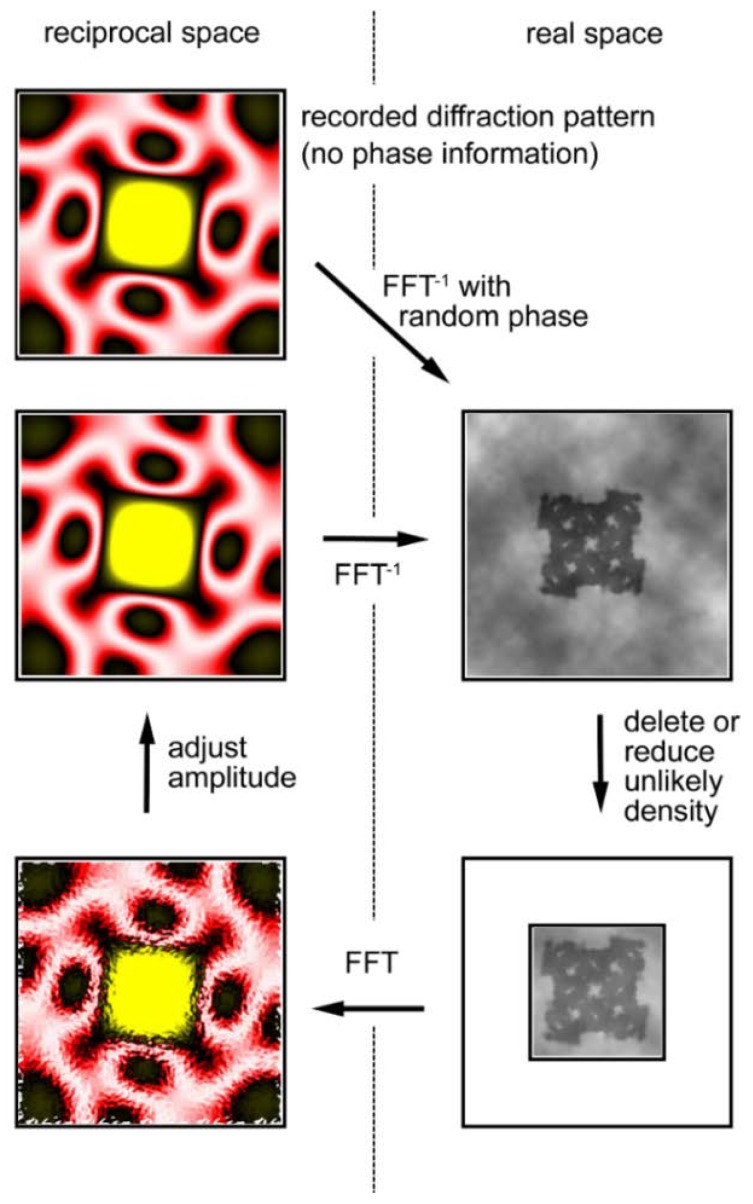
Iterative phase-retrieval algorithm (oversampling method). The recorded diffraction pattern from a specimen (in reciprocal space, its phase information is missing) is given a random set of phases (**Top left**). Then the pattern is subjected to inverse Fourier transformation (FFT^−1^) to obtain the real-space image (electron density distribution) of the specimen (**Top right**). This is incorrect because of the incorrect set of phases and it has densities in unlikely positions. Usually the approximate size of the specimen is known and we assume this area is likely to contain the specimen. This area is called a support (the smaller square) and is assumed to be somewhat greater than the expected size of the specimen. To successfully retrieve the correct phase information, the entire area of data collection (the larger square) must be at least 2× greater than the actual size of the sample. This is to ensure that the sampling frequency of data is at least 2× finer than the Nyquist frequency, which is defined as f = 1/s, where s is the size of the specimen (This is why it is called the oversampling method). Next, the densities that fall outside the support are reduced either to zero (error-reduction algorithm) or to a small fraction of the obtained densities (hybrid input-output algorithm or HIO algorithm) (**Bottom right**). After this, a Fourier transformation is applied to this corrected image to obtain a new diffraction pattern (**Bottom left**). This has a set of phases that is expected to be more correct than the initial set. By retaining this set of phases, the amplitudes are adjusted to the recorded values (**Middle left**) and the calculations are repeated in a clockwise direction. If all the conditions are right, the image of the sample converges to the true one after hundreds to thousands of iterations. Based on Reference [62].

The calculation, illustrated in Figure 9, starts with the observed diffraction pattern to which random phases are assigned. By performing a reverse Fourier transformation, one obtains a real-space image of the object, but the obtained image is incorrect. Although one does not know the exact shape of the object (the final goal of the calculations is to know it), one usually has some crude knowledge about its morphology, e.g., its size. Then one notices that unlikely densities are generated in the image and these unlikely densities are either reduced or deleted. The Fourier transformation of this image (diffraction pattern) has a new set of phases that are expected to be more accurate than the initial set of random phases. The amplitudes of the calculated diffraction pattern are adjusted to the observed ones.

By repeating these cyclical calculations, one may finally restore the correct set of phases and restore the true image of the object. Among such iterative algorithms, the most widely used one is called the hybrid input-output (HIO) algorithm [59] in which unlikely densities are not forced to zero but to small positive values.

This type of algorithm is regarded as a kind of model fitting that is employed in many fields of science including protein crystallography. One compares the observed diffraction pattern and the pattern calculated from the determined structure and infers that the determined structure is correct when the error between the two is reasonably small.

The problems common to this kind of iterative model fitting algorithm include: (1) the success of fitting depends heavily on the goodness of the starting model, (2) a good signal-to-noise ratio is required for the diffraction pattern, and (3) the calculations tend to be trapped by local minima. The HIO algorithm is known to be efficiently trapped by local minima, so that this algorithm is often used in combination with other algorithms, such as a genetic algorithm, to find the global minimum [63].

**Figure 10:**
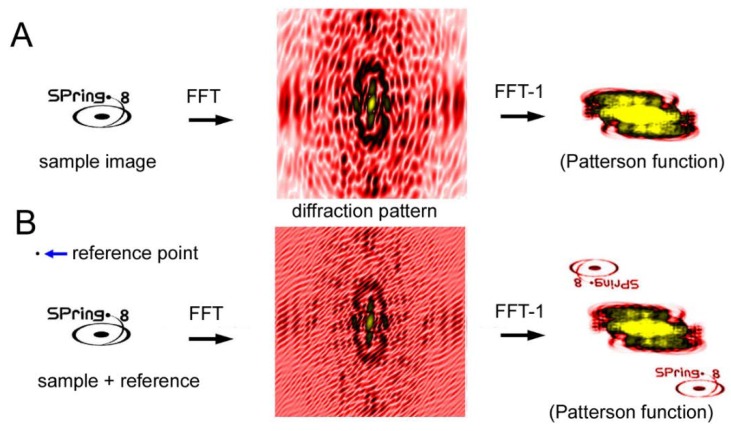
Principle of Fourier transform holography (FTH). (**A**) Diffraction from a sample (SPring-8 logo) and its inverse Fourier transformation (FFT^−1^) calculated by assuming that the phases are all zero. This results in what is called the Patterson function, which is an autocorrelation function of the sample. This is a real-space image but it is very difficult to imagine what the sample looks like by watching this function. (**B**) Diffraction from a sample with a reference point. The phase information is preserved in the Moiré pattern (caused by the interference between the sample and the reference) and, as a result, the real-space image of the sample is restored by performing inverse Fourier transformation. This is an experiment on a computer.

Another CDI technique is a holographic method and the representative type is called Fourier-transform holography (FTH) [64,65,66,67,68,69,70]. Unlike the iterative phase-retrieval algorithms as described above, phase information is recorded in the diffraction pattern itself. In the case of FTH, a small X-ray-scattering object (reference point) is placed near the sample. The X-rays scattered by the sample and those scattered by the reference point interfere with each other, which creates Moiré-like interference waves. The phase information is preserved in these waves. When an inverse Fourier transformation is performed on the diffraction pattern after assuming that the phase is zero everywhere, the image of the sample is restored (see Figure 10). The trick is that the inverse Fourier transformation of a diffraction pattern with zero phases (the Patterson function) is an autocorrelation function of the densities of the sample. If a reference point exists at a distance farther than the largest diameter of the sample, the correlation function between the sample and the reference point is the density distribution of the sample itself and it does not overlap with the rest of the autocorrelation function.

The merit of the FTH method is that the principle is simple and, in theory, it does not require any tricky algorithms but only one inverse Fourier transformation operation to restore the image of the sample. The demerit of this method is that one has to place a reference point at a certain distance away from the sample. The spatial resolution is determined by the size of the reference point. The larger the reference point, the stronger the interference is so that it is easier to retrieve the image at an expense of spatial resolution.

Both the iterative phase retrieval method and the FTH work fairly well with samples with high contrast such as metal nanoparticles, but it is more difficult to retrieve images from biological samples. In both methods, the achieved spatial resolution is currently of the order of 10 nm, which is unsatisfactory for biological specimens.

The application of these lens-less imaging techniques to conventional fiber diffraction patterns has not been tried extensively, but considering the rapid progress of algorithms and the improved performance of computers, we are expected to be able to retrieve real-space images of the muscle fine structure from the conventional diffraction patterns in the near future instead of analyzing the diffraction patterns in the reciprocal space we are currently completing.

**Figure 11:**
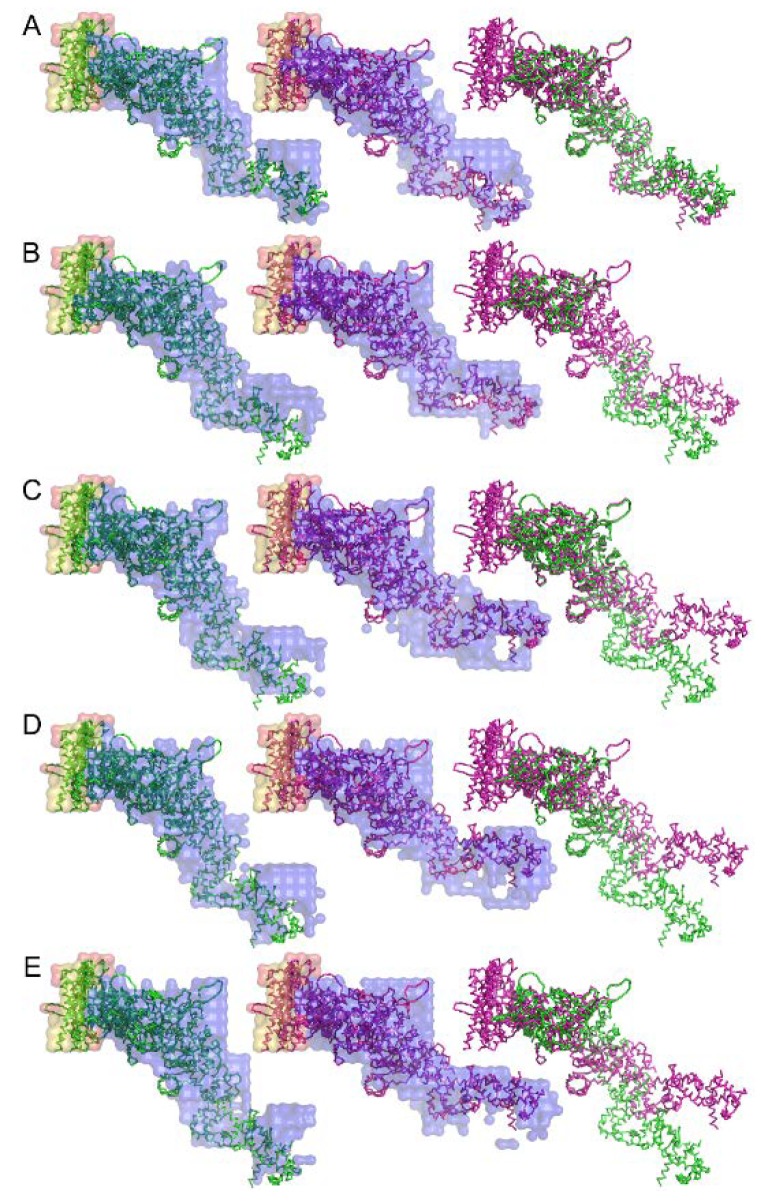
Structure of the complex between myosin S1 (subfragment-1) fragment and actin restored from diffraction patterns. The sources of S1 are: (**A**) rabbit fast skeletal, (**B**) rabbit cardiac/slow skeletal, (**C**) chicken gizzard smooth, (**D**) human nonmuscle IIA, and (**E**) human nonmuscle IIB. The green and purple amino-acid backbones are the best-fit structures for the electron-density envelope of the rigor and ADP-bound complexes, respectively. Taken from Reference [71].

An attempt to retrieve the 3-dimensional real-space structure of the actomyosin complex was done by iterative error reduction as early as 2007 before the days of XFEL [71]. Although at a low resolution, the 3-dimensional structure of the rigor complex between actin and myosin subfragment-1 from various sources and its structural changes upon binding of ADP were successfully restored (see Figure 11). Today we have much better knowledge and calculation power and we should be able to restore the real-space 3-dimensional image of the molecular machinery of muscle in a shorter calculation time and at a higher spatial resolution if we work hard to apply these emerging techniques to muscle research.

## 11. Conclusions

This review has explained how the bright and well-oriented synchrotron radiation X-rays can be utilized for muscle research especially for research of insect flight muscle. The techniques involved here include fast time-resolved diffraction recording and the recording from a single myofibril by using X-ray microbeams and rapid-freezing techniques. The interpretation of diffraction patterns still requires a proper understanding of diffraction theory and this can be an obstacle for general researchers to employ this powerful non-invasive technique. However, this situation will soon be changed when effective algorithms are available for restoring real-space images of specimens with ease.
